# A Simplified Amino Acidic Alphabet to Unveil the T-Cells Receptors Antigens: A Computational Perspective

**DOI:** 10.3389/fchem.2021.598802

**Published:** 2021-02-25

**Authors:** Raffaele Iannuzzi, Grazisa Rossetti, Andrea Spitaleri, Raoul J. P. Bonnal, Massimiliano Pagani, Luca Mollica

**Affiliations:** ^1^ Istituto Nazionale Genetica Molecolare INGM 'Romeo ed Enrica Invernizzi', Milan, Italy; ^2^ Molecular Oncology and Immunology, FIRC Institute of Molecular Oncology (IFOM), Milan, Italy; ^3^ Emerging Bacterial Pathogens Unit, Division of Immunology, Transplantation and Infectious Diseases, IRCCS San Raffaele Scientific Institute, Milan, Italy; ^4^ Department of Medical Biotechnology and Translational Medicine, University of Milan, Milan, Italy

**Keywords:** antigen recognition, receptor-peptide interaction, molecular mechanisms of adaptive immunity, ligand rational design, T-cell receptor (TCR)

## Abstract

The exposure to pathogens triggers the activation of adaptive immune responses through antigens bound to surface receptors of antigen presenting cells (APCs). T cell receptors (TCR) are responsible for initiating the immune response through their physical direct interaction with antigen-bound receptors on the APCs surface. The study of T cell interactions with antigens is considered of crucial importance for the comprehension of the role of immune responses in cancer growth and for the subsequent design of immunomodulating anticancer drugs. RNA sequencing experiments performed on T cells represented a major breakthrough for this branch of experimental molecular biology. Apart from the gene expression levels, the hypervariable CDR3α/β sequences of the TCR loops can now be easily determined and modelled in the three dimensions, being the portions of TCR mainly responsible for the interaction with APC receptors. The most direct experimental method for the investigation of antigens would be based on peptide libraries, but their huge combinatorial nature, size, cost, and the difficulty of experimental fine tuning makes this approach complicated time consuming, and costly. We have implemented *in silico* methodology with the aim of moving from CDR3α/β sequences to a library of potentially antigenic peptides that can be used in immunologically oriented experiments to study T cells’ reactivity. To reduce the size of the library, we have verified the reproducibility of experimental benchmarks using the permutation of only six residues that can be considered representative of all ensembles of 20 natural amino acids. Such a simplified alphabet is able to correctly find the poses and chemical nature of original antigens within a small subset of ligands of potential interest. The newly generated library would have the advantage of leading to potentially antigenic ligands that would contribute to a better understanding of the chemical nature of TCR-antigen interactions. This step is crucial in the design of immunomodulators targeted towards T-cells response as well as in understanding the first principles of an immune response in several diseases, from cancer to autoimmune disorders.

## Introduction

The adaptive immune system has the role of regulating a complex series of cellular and molecular responses to external menaces. Unlike the innate immune system, tailored to the identification of general threats, the adaptive immunity is activated by exposure to pathogens, and uses an immunological memory to learn about the threat and enhances the immune response accordingly. Lymphocytes are the cells in charge of adaptive immunity and they are grouped into two types: B cells are mainly responsible for the production of antibodies, T cells can either stimulate B cell activity or directly kill cells that are infected or malfunctioning (Pancer and Cooper, 2006).

The immune response is activated by small molecules (mainly short peptides with a well-defined structure) called antigens. T cell receptors (TCR) can only recognize antigens when bound to Major Histocompatibility Complexes (MHC), membrane-bound surface receptors of dendritic cells, and macrophages generally referred to as antigen presenting cells (APC). To make sure that T cells will perform properly once they have matured and have been released from the thymus, they undergo two selection processes, a positive selection and a negative selection. Positive selection ensures that T cells are capable of binding *via* TCRs only self-MHC molecules. Negative selection tests the binding capabilities of CD4 and CD8 receptors specifically on APCs to check the self-tolerance, e.g., ideally a T cell that only binds to self-MHC molecules presenting a foreign antigen. At the end of the selection process three types of mature T cells are left, i.e., Helper T cells (Th), Cytotoxic T cells (Tc), and T regulatory cells (Treg), characterized by a different physiological role and different receptors. Among them, Treg cells, which are physiologically engaged in the maintenance of immunological self-tolerance and immune homeostasis (Sakaguchi et al., 2008; Josefowicz et al., 2012), are potent suppressors of effector cells and are therefore involved in tumor development and progression by inhibiting antitumor immunity (Nishikawa and Sakaguchi, 2010; Haga-Friedman et al., 2012; De Simone et al., 2016), hence raising considerable interest as targets for the future development of anticancer drugs and therapies and the study of their receptors and mechanism is one of the newest frontiers in oncology.

At a molecular level, TCRs are expressed by four distinct genes (Tcra, Tcrb, Tcrg, Tcrd) that are rearranged in a dimeric form (αβ chains) during intrathymic T cell development. This causes the nearly limitless recombination of the genes that encode for T cell receptors and, at the same time, a lot of binding diversity. Theoretical numbers for human TCR diversity ranges from around 1,000 clonotypes, but the actual estimated TCR repertoire is ≈100 in humans (Villani et al., 2018). Such diversity is only reflected by the high variability of sequences of three loops of the TCRs, i.e., CDR1, CDR2, and CDR3 that are the only regions of the receptors that are able to interact with the MHCs and the structured-upon-binding antigen, whereas the overall fold and sequence are retained. Unlike antibodies, TCRs generally have low affinity for ligands (K_D_ ∼ 1–100 μM), which has been speculated to facilitate a rapid scanning of peptide-MHC (pMHC) compatible with positive selection (Birnbaum et al., 2014).

Structural studies of TCR-pMHC complexes have revealed a binding orientation where, generally, the TCR, CDR1 and CDR2 loops make the majority of contacts with the tops of the MHC helices while the CDR3 loops, which are conformationally malleable, primarily engage the peptide presented in the MHC groove (Garcia and Adams, 2005; Rudolph et al., 2006) (See Supplementary Figure S1). Moreover, some specific positions are well documented as being crucial for the recognition and binding of the specific components of the complex. In particular, focusing on MHC-II due to its central role in governing immune-oncological response and chronic inflammation (Painter and Stern, 2012), the so called P3, P5, and P8 positions along the antigen (progressively ordered from the N terminus to the C terminus) have a dominant role in the recognition of the CDR3 loops and consequently of the specific TCR, whereas the residues in position P1, P4, P6, P9, and P10 are crucial in regulating the interaction with the MHC-II antigen binding cavity mainly *via* backbone (antigen) –sidechain (MHC-II) interactions. Therefore, due to the large number of clonotypes (i.e. a unique nucleotide sequence that arises during the gene rearrangement process) of TCR encoded by the human genome, T cell cross-reactivity is expected to cover an enormous number of pathogen peptides presented on the cell surface of APCs (Villani et al., 2018). Indeed, given that the calculated diversity of potential peptide antigens is much larger than TCR repertoire diversity, TCR cross-reactivity appears to be a biological imperative (Mason, 1998; Wooldridge et al., 2012). In this respect, it is worth noting that the vast majority of antigens share sequence homology (Reiser et al., 2003; Adams et al., 2011).

Characterization of the T cells’ mediated immune response at the molecular level has benefited from recent advances in RNA sequencing (RNA-seq). Several groups have reported tools for TCR or Immunoglobulins (Ig) repertoire extraction from bulk RNA-seq or dedicated single-cell TCR enrichment protocols (De Simone et al., 2018). Since transcriptome sequencing has become routine in both basic and clinical studies (and TCR-antigen-MHC complexes are quite difficult to isolate and structurally characterize), it could serve as a source of functionally relevant information on immune receptor hypervariable region CDR3s repertoires (De Simone et al., 2018). The conservation of TCRs overall fold allows modelling of loops to highlight and characterize the subtleties of antigen recognition. Efficient computational solutions have been proposed in the last years (Gowthaman and Pierce, 2018), but the quest for discovering the biologically active antigens still remains open and of paramount importance in vaccine design, autoimmunity, and T cell therapies for cancer.

Peptide libraries are the most direct method to assess the effect of molecular details of antigens on the T regulatory cells (Bozovičar and Bratkovič, 2019) due to their superiority in specific cellular receptor targeting, stability at room temperature, good tissue permeability, lowering toxicity potential, and occurrence of off-target effects. A reiterative chemical modification approach can be honed for the development of peptide therapeutics with improved properties. Exploiting evolutionary principles in the laboratory by constructing and screening large peptide libraries can yield new lead compounds with the desired traits. Screening chemically synthetized peptides involves the libraries’ incubation with a fluorescently labelled soluble target or target-coated magnetic beads followed by flow cytometry-based systems (Stratis-Cullum, 2015) or magnetic separation (Stratis-Cullum, 2015), respectively. Pooled chemically synthesized peptide libraries have been successfully used in this field, leading however to an estimate of ∼10^6^ different agonist peptides in mixtures containing ∼10^12^ different molecules (Wilson et al., 2004; Wooldridge et al., 2012), offering an example of how time consuming and costly such a characterization could be (moreover, solely based on bulk stimulatory ability of peptides). Conversely, the main bottleneck of a cellular approach is a transformation step needed for delivering a DNA library into host cells, providing transcriptional and translational machineries for gene expression (Dell et al., 2010). The advantage of these methodologies is evident as well as the drawbacks related to the combinatorial nature of the technology and its costs.

The problem complexity can be reduced using a simplified library based on the contraction of chemical variability of the natural series of amino acids. This procedure is generated by clustering amino acids based on their relative similarity and brings forwards a reduced numerical dimensionality of the problem (Murphy et al., 2000; Etchebest et al., 2007; Peterson et al., 2009). Moving from this principle, we have adopted a simplified chemical alphabet of amino acids (SCAA) constituted by six amino acids representative of the main chemical classes, of the whole family of natural amino acids and implemented an *in-silico* methodology with the aim of verifying its reliability within the framework of TCR-antigen interactions. We have hypothesized an experimental setting that moves from sequencing data (i.e., the CDRα/β sequences from single-cell targeted TCR sequencing) to a set of potentially antigenic peptides of relatively moderate size thorough the TCR modelling and their screening *via* rigid body docking of a full combinatorial series of peptides with SCAA based sequences. In particular, we have verified the reproducibility of a set of experimental benchmarks (i.e., deposited TCR - antigen - MHC-II structures) as the ability of a SCAA to reproduce the experimental result with a good degree of accuracy both in terms of amino acids class correspondence and TCR surface placement with respect to the original dataset. We thus demonstrated that such a simplified alphabet is able to correctly find the poses and chemical nature of original antigens within a small subset of ligands of potential interest that can even be easily translated back to the language of natural amino acids in an experimental setup. The newly generated library would have the advantage of containing the leading antigen plus other potentially antigenic ligands that would contribute to a better understanding of the chemical nature of TCR-antigen interactions, a crucial step in the design of immunomodulators targeted towards a T-cell response as well as gaining a better understanding of the first principles of immune response in several diseases.

## Materials and Methods (Methodological Framework)

**FIGURE 1:**
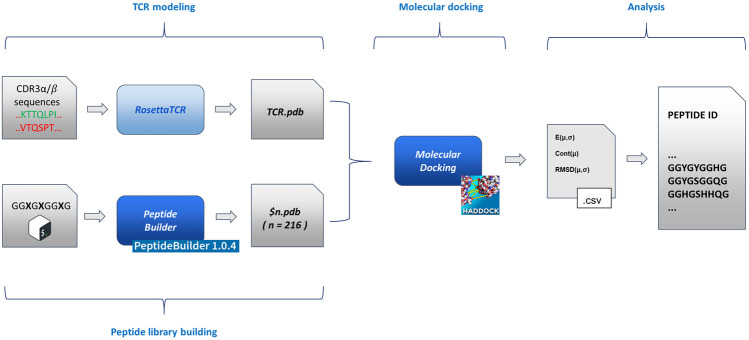
Flowchart of the method implementation. From left to right (as described in the text in the Methodological sketch section): TCR modelling and peptide building (based on PeptideBuilder) (Tien et al., 2013); rigid body docking of the TCR and peptides (based on HADDOCK) (Dominguez et al., 2003); statistical analysis and peptide list generation. The scheme implementation is commented in the Supplementary Material.

An exhaustive exploration of the chemical variability of the interactions between antigens and TCR would experimentally require, focusing on the variability of the sequence of the antigenic peptide (with an average length of 9/10 residues), the full combinatorial exploration of all the 20 natural amino acids alternating on the key positions of the antigen in all their possible combinations. Such a combinatorial approach, despite covering the entirety of the chemical variability constituted by the full set of natural amino acids, would lead to a time consuming and costly setup. For this reason, here, we explore the possibility to study the role and the nature of antigens involved in the immune response process by means of a reduced chemical space that would be efficiently mimicking the fundamental interactions that govern the TCR-antigen interactions and recognition. Such a chemical space can be thought of as being constituted by some amino acids that possess a sidechain averagely representative of an entire class of amino acids, to which it belongs, characterized by defined chemical properties (charge, aromaticity, etc.). This simplification of the chemistry involved in protein-protein interactions would lead to a dramatic reduction of the dimensionality of the experimental problem. To support this hypothesis at the atomic level, we developed and implemented a pipeline that moves from the TCR sequencing data and eventually generates a pool of antigenic peptides, through molecular docking of modelled TCR α/β and a series of structured peptides (i.e., with the same extended structure adopted after the binding to the MHC) associated to a statistical analysis of its results. In particular, we benchmarked our pipeline, testing it against a series of experimentally documented interactions (i.e., available crystallographic structures). The pipeline (Figure 1) is constituted by four blocks:

1. *Choice of TCRs benchmarks and CDR3α/β loop modelling*: the sequences of (at least) CDR3 α and β chains, as potentially extracted from the RNA sequencing experiment, are used to model the structure of the receptor under investigation;
2. *Peptide library construction and simplified chemical alphabet*: the sequences as well as the dihedral Φ/Ψ angles are decided on the basis of the desired representation of the chemical space relative to the interaction under investigation;
3. *Restrained rigid body docking*: the full set of peptides that constitute the library is docked on the surface of the TCR in order to accommodate them according to experimentally derived restraints, energetic, and contact criteria;
4. *Results inspection and analysis*: the outcome of the docking calculations is analyzed to retrieve information about the peptide-receptor interaction on the basis of the protein-peptide binding energy, of the contacts and of the root mean square deviation (RMSD), with respect to the original reference structure.

Details about the implementation of the flowchart are reported in the Supplementary Material.

## Choice of TCRs Benchmarks and CDR3α/β Loop Modelling

To correctly inspect the complete chemical recognition system that governs the interaction between antigen presenting cells and T cells, we decided to select only a set of completely resolved complexes formed by T cell receptors (TCR), the major histocompatibility complex of type II (MHC II), and the antigen comprised between the two. The number of completely resolved three-bodies complexes in the Protein Data Bank is relatively low with respect to other systems, i.e., 64 unique complexes structures were deposited at the end of January 2020: moreover, about 25% of them were redundant in terms of the amino acid sequence of the antigen and/or of the TCR sequence. We therefore decided to select seven complexes: 1zgl, 2ian, 3mbe, 3t0e, 5ksb, 6cqr, and 6dfx (Table 1). In particular, we selected the ones that seemed to be the most representative of the inner variety of the group of available complexes in the PDB considering two characteristics at the same time: the heterogeneous overall length of the antigen, ranging from seven to 15 amino acids; a reasonable variety in terms of sequence and length of the CDR3α/β TCR loops.

**TABLE 1:** Summary of energies and contacts.

PDB	Resolution (Ǻ)	Reference triad	E_i_ ref (kcal mol^−1^)	C_i_ ref	E_i_ min (kcal mol^−1^)	E_i_ max (kcal mol^−1^)	C_i_ min	C_i_ max	Translated triad	E_i_ (kcal mol^−1^)	C_i_
1zgl	2.8	N V R	−12.6	298	−20.1	−10.8	241	329	Q V H	−18.8	282
2ian	2.8	T Q K	−14.7	273	−20.7	−13.0	206	283	S Q H	−18.3	240
3mbe	2.9	G D R	−6.4	360	−14.4	−5.4	242	345	V D H	−13.4	282
3t0e	4.0	A G P	−8.2	264	−16.0	−7.3	235	312	V V V	−11.3	275
5ksb	2.9	Q F Q	−17.5	309	−17.6	−7.8	259	342	Q Y Q	−15.1	335
6cqr	3.0	Y R Q	−7.2	108	−19.1	−7.1	185	327	Y H Q	−19.1	185
6dfx	2.0	Y V E	−8.2	335	−16.4	0.4	274	337	Y V D	−5.4	335

List of benchmarks used in the present article including the PDB ID of the benchmark, the resolution of the original crystal structure, the original triad of amino acids and its translation in the SCAA, the extreme values (i.e., minimum and maximum) of the region centered on average values of C_i_ and E_i_ and considered within an interval defined as ±σ, the values of E_i_ and C_i_ of the reference original triad of amino acids and those computed for the translated triad (respectively expressed in kcal mol^−1^ and as pure numerical values).

Adopting the point of view of anyone who is experimentally investigating these systems, with access to sequencing data only (i.e., CDR3α/β TCR loops variable regions), we have also modelled the original sequences (i.e., the ones that correspond to the deposited structures) using the Rosetta TCR software (Gowthaman and Pierce, 2018) as reported in the pipeline flowchart (Figure 1). Due to the experimental templates-based algorithm of the software and the extremely conservative nature of TCRs’ structure, we eventually obtained almost completely superimposable structures of the receptor (maximum backbone RMSD ≈0.5 Å, data not shown). We therefore decided to use the native structures.

## Peptide library Construction and Simplified Chemical Alphabet

Since the conservation of Φ/Ψ backbone dihedral angles of antigens is well documented in the literature (Painter and Stern, 2012), we extracted from the selected TCR-antigen-MHC II complexes the solid angle pairs necessary for the construction of a rigid antigen geometry with results already favorable for the interaction with CDR3 loops of TCRs. The complete list of Φ/Ψ backbone dihedral angles, alongside the template orientation of antigen-TCR interactions, is reported in the Supplementary Material.

**FIGURE 2:**
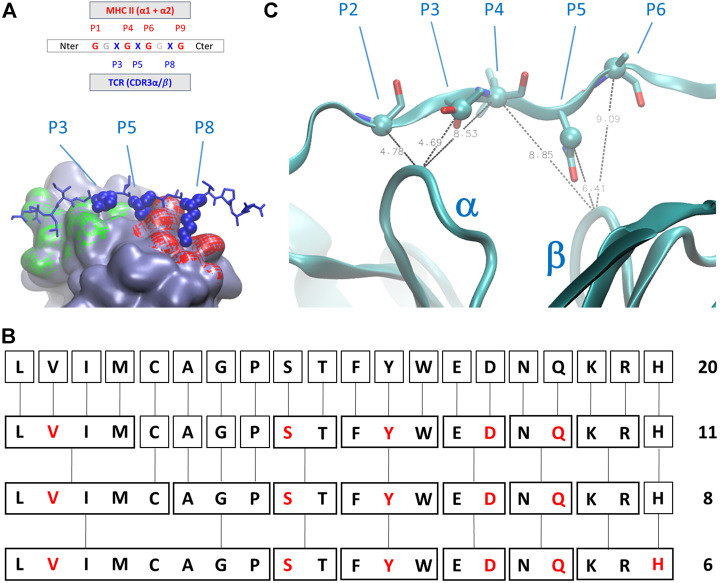
**(A)** Schematic representation of the amino acids sequential nomenclature along the sequence of the antigen; a global 3D geometrical view of the interaction surface of TCR chains α (green) and β (red) with antigens is reported alongside its nomenclature; **(B)** simplified chemical alphabet of amino acids (SCAA) adopted in the present work, with the derivation from 20 amino acids scheme to the 6-letters alphabet (in red) adopted in the present work according to the clustering principles presented in (Murphy et al., 2000); **(C)** ambiguous restraints used for docking calculation of the antigen pose on the surface of T-cell receptor (TCR); the distances are expressed in Angstrom, the Cα and Cβ atoms are highlighted as spheres and CDR3 α and β chains are indicated. The molecular graphics has been realized using Visual Molecular Dynamics 1.9.3 (Humphrey et al., 1996).

The geometry of complexes formed by TCR, antigens, and MHC-II revealed that the number of antigen residues that are directly involved in the modulation of the interaction between the TCR and the MHC-II is restricted with respect to the total number of interactions and contacts between the antigen itself and the two major partners in the complex formation. In detail (Figure 2A), numbering the residual position from the C-terminus, the positions P1, P4, P6, P7, and P9 are occupied by residues that directly interact with the MHC-II cavity formed by helices belonging to α and β subunits, whereas the amino acids in position P3, P5, and P8 are characterized by sidechains that protrude toward the pockets formed by the CD loops of TCR (Painter and Stern, 2012).

The latest three positions along the antigen constitute the most relevant and challenging part of the problem. Indeed, the interactions between the antigen and the MHC are mainly constituted by direct contacts between backbone antigen atoms and sidechains of MHC II (Painter and Stern, 2012), and complimentary anchor/pocket interactions are not an absolute requirement for MHCII/peptide affinity and immunogenicity (Ferrante, 2013). The residues in P1, P4, P6, and P7 can have a broader spectrum than the ones in P3, P5, and P8 (from now on, the immunogenic triad, ImmT) and are therefore the ones really worth a more detailed investigation.

We then decided to adopt a simplified chemical alphabet of amino acids (SCAA for short) for sampling the chemical nature of the interactions occurring between the antigen and the TCR ImmT. To this aim, we decided to sample the interactions between the TCR cavities exposed towards the MHC-II and a selection of six amino acids as representative of chemical “classes” (Figure 2) largely based on the, quite common, BLOSUM scheme (Murphy et al., 2000) and on a reasonable assumption about the size of the set of representative amino acids (Solis, 2015):

1. Protonated histidine (His, H) has been selected for a positively charged moiety, whereas aspartate (Asp, D) has been chosen for a negatively charged moiety. Both these residues have been selected according to a compactness criterion, to avoid any artefacts, during the following docking procedure, due to an arbitrary selection of long and naturally flexible sidechain conformations. Moreover, the local extracellular environment in many immune responses is slightly acidic (Boedtkjer and Pedersen, 2020), thus justifying the usage of protonated histidine as a spy moiety;
2. We adopted the same principle for the choice of serine (Ser, S) and glutamine (Gln, Q), for an uncharged polar group (with the possibility of being deprotonated) and for an uncharged polar group (with the possibility of being protonated), respectively. Valine (Val, V) has been chosen as representative of a hydrophobic moiety, whereas for the aromatic moiety we have chosen tyrosine (Tyr, Y) to reduce the chemical complexity of the library especially in terms of the number of degrees of freedom.

SCAA aside, every residue in different positions with respect to the ones of the ImmT has been occupied by a glycine (Gly, G), drastically diminishing the possibilities of unwanted interactions between the triad of amino acids essential for mapping the TCR-antigen recognition and having previously ensured the maintenance of the correct antigen geometry. In this way, the peptides will always have a sequence in the form of GGXGXGGXG, where X corresponds to a residue that will be replaced by one belonging to the SCAA. A global scheme of translation is reported in Figure 2. It is worth noting that this choice is not a unique one that could have been done for a simplification of the variety of amino acids, but this scheme probably represents one of the best compromises between dimensionality reduction of the chemical space of antigens and the representativeness of fundamental interactions in protein structures according to the studies mentioned above.

The peptide library used for the present work has been generated using the program PeptideBuilder 1.0.4, a Python library for the generation of model peptides (Tien et al., 2013), starting from the aforementioned list of solid angles and combinatorically generating the complete list of all the available peptides using the simplified chemical alphabet discussed above. This resulted, in combination with the SCAA, in a library of 216 structured peptides (SCAA-Lib) that served as a starting pool for sampling the interaction between the TCR and the antigen alongside the interaction of the former with MHC-II.

## Restrained rigid Body Docking

For every peptide of the SCAA-Lib, a rigid body docking simulation has been performed using the software HADDOCK 2.0 (High Ambiguity Driven protein-protein Docking) (Dominguez et al., 2003), a scripting system that makes use of biochemical and/or biophysical interaction data such as chemical shift perturbation data, fluorescence experiments, mutagenesis data, and many others. This information is introduced as Ambiguous Interaction Restraints (AIRs) to drive the docking process, which is processed by the molecular mechanics’ engine, CNS (Brünger et al., 1998) on top of which HADDOCK is built. An AIR is defined as an ambiguous distance between all residues shown to be involved in the interaction.

In the present case, despite no direct and/or new experimental information being accessible, we considered the extreme similarity among the whole series of deposited structures of TCR-antigen-MHC II to drive the docking of model peptides on the surface of TCR. Due to a possible bias introduced by sidechain rotamers selected during the library construction phase, we set up AIRs for these systems selecting some average distances between Cα and Cβ atoms of the antigen and Cα of the central residues of CD3 loops. In particular, only four AIRS have been imposed during the docking calculation: 1) the distance between the residue P2 Cα, P3 Cβ, or the P4 Cα atoms and the Cα atom of the central residue of the CD3α loop; 2) the distance between the residue P4 Ca, P5 Cβ, or the P6 Cα atoms and the Cα atom of the central residue of CD3β loop (Figure 2C). The distance ranges have always been set up between 4 Å and 8 Å. This strategy ensures that the placement of peptides are very close to the exposed surface of CD3α/β region of the TCR and a reasonable pose search on the basis of steric hindrance of amino acids. For every model peptide of the library, we generated 100 poses. Details about the docking procedure are reported in the Supplementary Material.

## Results Inspection and Analysis

We considered all 100 poses generated for each model peptide as representative of the binding mode and the intrinsic dynamics of the TCR-antigen interaction: this choice has also been supported on the basis of the relatively low standard error of the average of the protein-peptide interaction energy computed for each complex using CNS, which is always comprised between 1% and 5% of the value (see further). We therefore mapped the phase space of the possible interactions between the model peptides on the basis of all 100 configurations obtained for each peptide. Aside from the interaction receptor-peptide binding energy, we also considered the atomic contacts between the peptide and the receptor for the characterization of the complexes, which recently emerged as a good estimator of the binding in protein-protein interactions in correlation with experimental affinities as described in studies of benchmark complexes of different sources and nature (Vangone and Bonvin, 2015). The usage of a second parameter for the discrimination/grouping of ligands is also helpful to avoid any issues arising from an excess of the sensitivity of the binding energy to all the approximation introduced in the screening pipeline at any level. This parameter has been computed using the utility implemented in the program GROMACS (Berendsen et al., 1995; Abraham et al., 2015) due to the popularity of this software among computational chemists’ interested in biological macromolecules. Details about these analyses are reported in the Supplementary Material.

## Results and Discussion

The pipeline we ideated stemmed from many pre-existing and documented building blocks from many fields and applications in the area of biophysical computational chemistry, glued together by the central idea that a protein surface can accommodate a variety of ligands that share one or many common features. Indeed, the surface that is able to accommodate antigens, has already been conformationally modelled by its major partner MHC in the immune response process at the molecular level.

In a way similar to a drug discovery screening procedure, our problem can be seen as the screening of many ligands that share a common scaffold. In this respect, the proposed method has the advantage of being faster than an extensive screening. Moreover, one major advantage of the presented procedure is its potential use for any similar problems that can be encountered in the world of protein-protein interaction, i.e. all the interactions between one target protein and many polypeptides that share a common structural motif but display a big variability in terms of their (at least) local chemical nature (e.g., chromatin modifications (Zhang et al., 2016), as post-translational modifications influence signalling (Duan and Walther, 2015)). This approach has the advantage of presenting almost no bias dependent on the internal entropy of the ligands (due to their rigidity) and consequently relies on the evaluation of the binding energy as the only contribution to the interaction.

**FIGURE 3:**
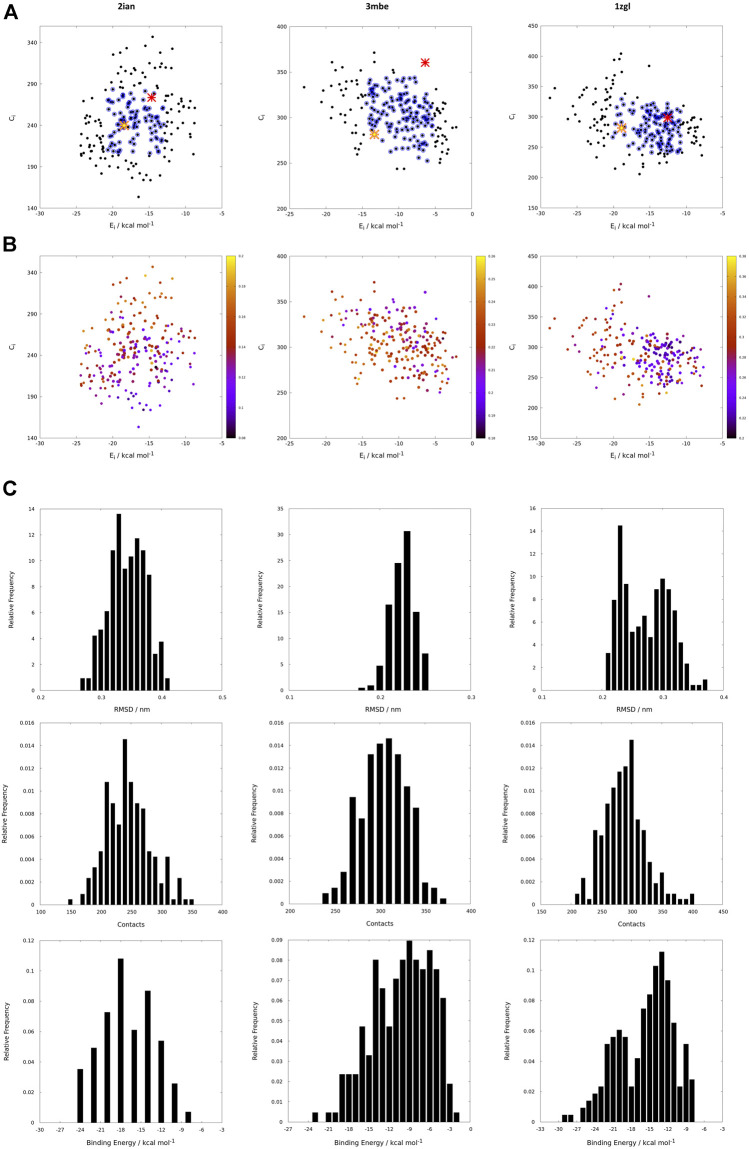
**(A)** Energy vs contact maps relative to the benchmarks 2ian, 3mbe and 1zgl. The other benchmarks used in the article are reported in Supplementary Figure S2. Every black dot represents the average of 100 poses for a single peptide; the blue dots correspond to values of energies, E_i_, and contacts, C_i_, that fall within intervals of ±σ centered with respect to average values of the plotted property; the red cross corresponds to the original triad of amino acids in positions P3, P5 and P8; the yellow star corresponds to the translated triad of amino acids in positions P3, P5, P8; **(B)** Energy vs contact maps relative to the benchmarks 2ian, 3mbe and 1zgl: every dot is colored according to the RMSD with respect to the original experimental position. The other benchmarks used in the article are reported in Supplementary Figure S3; **(C–E)**; E_i_, C_i_ and RMSD distribution of the peptides for 2ian, 3mbe and 1zgl benchmarks. The other benchmarks used in the article are reported in Supplementary Figures S4–S6.

If the protein-ligand binding energy can be seen as the foremost important parameter in the assessment of the interaction between the two partners, we preferred to also use two other parameters for its evaluation; the number of contacts was used as a predictive parameter of the goodness of binding search result and the root mean square deviation (RMSD) with respect to the backbone orientation of the original peptide pose of the benchmark used as a downstream control of the good quality of the fit. Without prior knowledge of the experimental outcome, all the interactions can be mapped using only interaction binding energy (E_i_) and contacts (C_i_) as coordinates for the two-dimensional representation of the interactions between the TCR and the model peptides (Figure 3). Using this representation, every point on such a map (reported in black in Figure 3) represents the coordinates of a single peptide averaged over 100 poses in both dimensions for a single docking run. The ambiguity driven nature of the generation of docking poses in HADDOCK, leads to a set of very close orientations of the peptides on the surface of the proteins. The values therefore implicitly represent a weighted average of the binding energy value over all the poses that account for their relative populations.

The most probable and logical scenario is the one reported in Figure 1A, using the benchmark structure 2ian as an example. In this case (that is present also in the benchmarks 3t0e, 5ksb and 6dfx, see the Supplementary Figure S2) the distribution of energies follows a rather unimodal distribution, which could be either symmetric or skewed (see also Supplementary Figure S5). The same trend is clearly exhibited by the distribution of contacts, with the exception of the 6dfx structure (see also Supplementary Figure S6) that seems to exhibit a slight bimodal trend. This similar behaviour for all these systems suggests that the interactions between the TCR surface and the antigenic peptide, despite the chemical differences along the complete series of ligands, cluster around central values of contacts and energies. This kind of distributions led us to analyze them in terms of ensembles of most probable binders, suggesting the existence of a core region of the phase space of the binders comprised within an interval of ±σ (standard deviation of the mean) around the central average values (represented with blue circles in the Figure 1).

This subspace constitutes, in our opinion, a pool of the best binders among the SCAA-Lib for every single TCR under investigation, on the basis of contacts and interaction potential energy. If in principle this should constitute the goal of a standard docking procedure and consequently indicate the ligands that best fit the interaction surface of the receptor, some *caveats* are necessary for connecting our calculations to a chemically and biologically meaningful result. The first one is related to the ability of the proposed methodology to detect, among all the best binders, the one that really corresponds to the original triadic sequence translated into SCAA. In order to verify this aspect of our calculations, we monitored both the untranslated (red star) and the SCAA translated (yellow star) triad using the aforementioned maps. In this first group of benchmarks, both peptides fall within the boundaries of the best binders and the translated triad is located almost exactly in the middle of the distribution, thus indicating that both peptides share a chemical similarity and consequently belong to the same phase subspace of the TCR-peptide interactions (see also Table 1). Considering the 3t0e, 5ksb, and 6dfx benchmarks (Table 1, Supplementary Figure S2) the translated triadic sequence occupies a central position with respect to the original sequence in both the E_i_ and C_i_ phase space dimensions. Conversely, the original triads are located at the margins of the best binders’ distributions, i.e., with an average correct placement of the C_i_ values only for 3t0e and 5ksb and of the E_i_ for 6dfx. These results demonstrate, in the frame of reduced chemical alphabet, the contextual superiority of the translation in reconstructing experimental results within the presented pipeline. Such behaviour is intimately connected to the adopted docking method; the peptide poses are generated using restraints that to some extent mimic the presence of the MHC that shapes the TCR surface. The structure of the TCR is considered rigid, hence no surface adaptation drives optimal binding with energy not necessarily located in the minima of the HADDOCK score distribution. Intriguingly, however, the placement of the translated reference triad, around the average of the graph and not in the lowest energy and/or highest contact regions, is in agreement with some biophysical data indicating that TCRs generally have low affinity for ligands (K_D_ ∼ 1–100 μM), which has been speculated to facilitate rapid scanning of peptide-MHC complexes, supported by the idea that the best binders are not the ones that are more tightly bound (Rudolph et al., 2006).

The Cα atoms RMSD values range from less than 1 Å to 3 Å (Table 1, Figure 3 and Supplementary Figure S3) with a minor percentage of the structure displaying an RMSD comprised between 3 Å and 4 Å. These values indicate that this result is, in general, in fairly good agreement with the experimental reference structure (Lensink and Wodak, 2013), also considering the relatively low resolution of the crystal structures (Table 1). They also suggest that encounter complex formation dynamics could have a role in adapting the poses of the three partners together, as proposed in the past (Ding et al., 1999; Hoffmann et al., 2017; Wieczorek et al., 2017). This aspect is the basis for the second important caveat; the results obtained with the presented procedure take into account only the chemical nature of the TCR surface portion that interacts with the antigen, probing such interaction in a pure energetic and geometric fashion without considering the presence, in the biological context, of the MHC-II. If this is not primarily relevant from a pure physical-chemical point of view in terms of the direct antigen-receptor interaction, it should be considered with care in the biological context of the complex formation. For this reason, the chemical space sampling scheme we propose can be thought of as a good method to probe for the primary antigen-receptor interactions, but it can include dynamics correlated to the chemical nature of the ligands proposed by the MHC—itself quite a dynamical actor of this interplay (Painter and Stern, 2012; Fodor et al., 2018).

The importance of the biological role of the MHC-II in setting up the correct geometry for the binding of the antigen to the CDR3 regions of the TCR is highlighted by the completely different scenario presented by the 3mbe benchmark (Figure 3). In this case the RMSD, with respect to the reference, is overall quite low but the original triad falls in a very peripheral region of the contact-energy map, thus suggesting that what is vehiculated in cells by the presence of MHC-II is very likely not the best accommodation of the peptide in terms of energy minimum and contact optimization. However, the relevance of local chemistry governed by sidechains’ nature is preserved when the translated peptide is used in place of the original one, with the former falling very close to the central region of the best binders. Hence, our method of reduction of chemical variability ensured the possibility of finding the triad of amino acids corresponding to the translation of the original antigen. The SCAA translated triad is located in a region that displays quite a uniform RMSD value distribution (≈2.6 Å), even if a bit higher than the average (≈2.2 Å). A similar result is obtained for the 6cqr benchmark (see Supplementary Figure S2,S3), with a more dramatic difference between the native and translated reference.

The results obtained on 1zgl (Figure 1C) have almost the same features displayed by 2ian and similar benchmarks. The remarkable difference is represented by the asymmetric profile of the RMSD values distribution. In this case two regions can be clearly identified in the RMSD values map, with the original triad belonging to the most populated “cluster” with an average RMSD of ≈2.5 Å and the translated peptide located in between the two main maxima identified both through E_i_ values and RMSDs. Considering that the overlap of the tails of the two separate distributions is big enough, we are still able to catch the essential chemical nature of the best peptide among the selected ones and, more generally, select a family of triads that can be used to sample the receptor-antigen interaction phase space.

Interestingly, 1zgl reference publication (Li et al., 2005) reports an experimental demonstration of the TCR degeneracy using superantigens, i.e. peptides with substitutions at nearly all TCR-contacting positions that are still able to bind the receptor surface once they are correctly placed in the antigen-binding cavity of MHC. The original sequence of superantigens is (numbering from P1) FKLIXTYKZ, with P5 X = L/T/P, P7 Y = T/K/P and P9 Z = L/G. The translation of all the possible combinations of point substitutions leads, in our pipeline, to the generation of only two model peptides, which result from the replacement of the MHC anchoring residues with G and residues at TCR binding position P3 with V (from L), P5 with V/S (from L or P and from T respectively), and P8 with H (from K), leading to peptides GGVGVGGHG and GGVGSGGHG. The binding energy and contacts are −19.6 kcal mol^−1^ and 291 for GGVGVGGHG, −19.8 kcal mol^−1^ and 310 for GGVGSGGHG (see Supplementary Table S1), a result that places the two model superantigens in the leftmost region of the best binder’s family reported in the map in Figure 3 but still very close to the translated reference peptide (−18.8 kcal mol^−1^ and 282 contacts), in agreement with experimental results. For the sake of completeness and comparison, we have also simulated the pose of the untranslated triads (see Supplementary Table S1) that resulted closer to the reference triad than the translated correspondents. Despite the paucity of reference experimental data, this comparison reveals the ability of our method to interpret, at the level of local chemical environment, relative binding properties of peptides that share a similar spectrum of interactions.

The aforementioned results demonstrate the robustness and reliability of the idea behind the simplification of amino acids sidechain chemistry using only a few residues that are grossly representative of the main chemical physical characteristics of sidechains that mediate interactions between a peptide and a protein/receptor. In this respect, we demonstrated that the chemical space occupied by a triad of six selected representative residues (Asp, Gln, His, Ser, Tyr, Val) is able to correctly detect the leading interactions between an antigen and the surface of the TCR and, at the same time, to reduce the dimensionality of the problem. The reduced alphabet would require a triad 6 × 6 × 6 = 216 combinations of spy amino acids, whereas the complete set of natural amino acids would require 20 × 20 × 20 = 8,000 combinations, with a ≈40 times reduction. If this number has a great impact on the computational cost of the whole procedure, it also influences experimental activity, implying the possibility to reduce the dimension of libraries, the costs of experiments, and the operational times of screening. Moreover, such a subfamily can be expanded using the amino acids belonging to it and lead, apart from the original antigen, to the identification of cognate peptides that exert the same biological effect, thus heading the exploration of the effects of conservative mutations on both the binding strength of the system and the immunological response. In this respect, this approach can also be helpful in designing and optimizing therapeutic peptide vaccines (Malonis et al., 2020) against the TCR of interest. Last but not least, this family of triads can be used in a bioinformatics approach for the exploration of conserved or similar sequences in the protein realm (e.g., through BLAST search) to formulate hypotheses on the origin of antigens and on the biological mechanisms at several levels that contribute to the immune response *via* other partners in their biological context.

**FIGURE 4:**
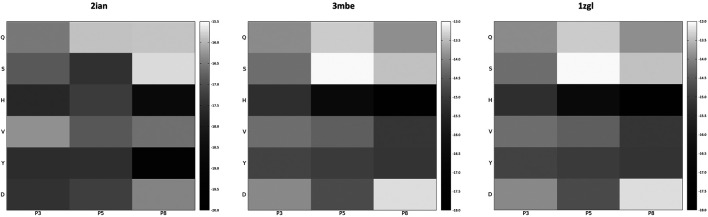
Energy residual content heatmaps for the benchmarks 2ian, 3mbe and 1zgl with respect to the SCAA class (vertical axis) and the three positions along the antigen sequence P3, P5 and P8 (horizontal axis). The other benchmarks used in the article are reported in Supplementary Figure S7.

If the dimensionality reduction already constitutes an advantage from several points of view, the optimal goal of a computational method of the presented type is the identification of the real antigens or, more generally, to restrict the number of combinations that could be used for further analysis or experiment. Unfortunately, the analysis of the positional frequency of the best binders for each benchmark (data not shown) reveals a quite homogeneous distribution, thus disallowing a clear statistical preference of few residue types for each position along the sequence that faces the TCR surface and, consequently, a clear indication of how to tailor the construction of a peptide library for screening even more. This is not completely surprising; the phenomenon of binding has a strong synergic nature and, in this case, again, it depends in the cellular environment on the presence of MHC-II that strongly conditions the final antigen placement on the surface of the receptor. However, an energetic positional analysis (Figure 4 and Supplementary Figure S7) can at least give some hints about a further refinement of the library. We have computed the average E_i_ content for every type of amino acid located at positions P3, P5, and P8 and graphed them in a heatmap (Figure 4) that visually allows the detection of some trends along the amino acid series or along the sequence. A much lower/higher residual energy content, referred to as the energy span of the series, should indicate that a given type of amino acid coupled to its position would contribute much more/less to the binding in the contest of the best binders’ series. Conversely, an almost uniform distribution along the series or along the sequence would suggest the absence of preferential choices. In general, this evaluation could, in principle, allow the exclusion of some groups of amino acids from the generation of the library in specific positions along the sequence of the antigenic peptide. The three cases reported in Figure 4 (corresponding to the ones presented in Figure 3) exemplify this principle; overall this approach correctly excludes some groups of amino acids in positions for which, experimentally, we know that an amino acid belonging to a completely different group is present. If this does not solve the central problem of *in silico* discovery of the correct antigen, it has the advantage of further reducing the complexity of the problem of experimental library design. For example, the 1zgl benchmark leads to the exclusion of Ser for the position P5 and of Asp in position P8. Using this restriction, the library would still include the translated triad Gln – Val – His (leading back to the original untranslated triad Asn – Val - Arg) and would contain 6 × 5 × 5 = 150 permutations, almost reducing one third of the original full combinatorial library and consequently reducing the original native full amino acids library size ≈ 60 times. The residual energy map of benchmark 6cqr (Supplementary Figure S6) suggests that a similar or even more dramatic reduction is possible. The maximum energetic content per residue is considered here and caution is used in treating minima; the most important contribution to the binding along the sequence is not considered as a restricting choice that would lead to wrong exclusions, i.e., a net choice of histidine in position P8 would exclude the experimentally obtained result. Based on these findings, a generally good principle for further reducing the size of the library seems to be the exclusion of positional global maxima (like it occurs also in the 2ian and 3mbe cases), without being tempted by any straightforward choices due to minima.

On the basis of our methodology, the overall complexity of a library of antigenic peptides is reduced only due to the chemical space manipulation of the residues that interact with the TCR surface. However, the quest for finding optimal/reduced combinations of amino acids still remains open on the MHC-II side, a topic for which several solutions have been proposed in the last decade, ranging from biochemical screening *via* libraries (Birnbaum et al., 2014) to machine learning (Barra et al., 2018). This problem, however, is beyond the aim of the present work, but it is worth noting that in principle the same reduced chemical alphabet we used for the TCR can be used to integrate the possibility of interaction of the antigen with MHC-II. The exact combination of amino acids can be combinatorically restrained on the basis of existing biochemical and structural information of the exact MHC-II involved in the immune response mechanism. Namely, the exact experimental knowledge of the MHC-II sequence *via* RNA sequencing experiments performed on the antigen presenting cells can be exploited and homology modelling can be performed with ease due to the great conservation of MHC-II sequences. Moreover, a relatively high variability and low relevance of the specific amino acid types belonging to the central portion of the antigen and facing the MHC-II antigen (Painter and Stern, 2012; Birnbaum et al., 2014) are documented as a consequence of the stabilization of the antigen-MHC-II complex by direct interactions between backbone antigen atoms and MHC-II sidechains. At the same time, the P2 position can be relevant for the stabilization of the antigen-TCR interactions (Birnbaum et al., 2014), requiring an efficient exploration and no particular restraints. P1 and P9 positions are usually among the most relevant anchoring points (Sant’Angelo et al., 2002) for the antigen to the MHC-II and they can be restrained, as recently demonstrated in one or two residues (Birnbaum et al., 2014). For these reasons we suggest, on the basis of our methodology and of the simplification we introduced in the biochemical alphabet for screening the antigens, to build a library of decamers ranging from position P-2 to position P9 adopting the SCAA (six residues per position) and to restrict, if possible, the combination to a couple of amino acids on positions P1 and P9. This scheme would generate a library of 2.5 × 10^6^ peptides (see Supplementary Figure S8 for a general dimensionality reduction scheme that descends from the presented method) that could, in principle, be used to grossly explore *in vitro* T cell responses to antigens positional chemical moieties with ordinary methods at an affordable price (Joglekar and Li, 2020), to select the most relevant binders on the basis of their biophysical and/or biochemical properties, and to expand their chemical spaces back to 20 amino acids alphabet and refine the hunt of the real antigens within a given biological context.

## Conclusions and Perspectives

In the last decade substantial efforts in chemically oriented research has provided atomic and molecular level details of the immune response, ranging from the connection between cancer and immunity to the subtle and often elusive nature of autoimmune diseases. In this sense, considerable attention has been drawn to one single but heterogeneous and variegate macromolecular complex that characterizes immune response by T-cells, the one formed by the T-cells receptor, or TCR, and the major histocompatibility complex, MHC-II. A key role in the formation of this complex as well as in the immunological response is played by the antigens, small peptide chains that mediate the effectiveness of the interaction, which have an extremely well conserved structure but an also rather elusive sequence. Our contribution to the mechanistic and molecular/atomic level study of the immune response, is the identification of a small set of residues (Asp, Gln, His, Ser, Tyr, Val) that can be used as model chemical moieties for the study of the interaction between the antigen and the hypervariable complementarity-determining regions (CDRs) of the TCR. This group of six residues can be used to build simplified libraries to be exploited *in vitro* (and, potentially, also *in vivo*) for the identification of the molecular determinants of interactions that govern the T cell’s immune response and, at the same time, a pool of peptides from which a new generation of ligands can be obtained and tested again with a full amino acid alphabet, encompassing a family of possible antigens related to specific biological contexts. More generally, we also provided a methodological framework that we developed *ad hoc* to demonstrate the effectiveness of reduced dimensionality of the permutations problem within a library. Such a method is based on well-known principles and software integrated with our own code and can be applied to several protein-protein interaction problems that rely on the existence of given reference experimental structures and on the necessity to inspect the effect of ligand amino acids permutations on the complex formation. We envisage for our method a future perspective that encompasses an experimental verification of the alphabet reduction principles we formulated in the present work and its application on different systems, to verify its universality and to investigate its first principles in more depth.

## Data Availability

The raw data supporting the conclusions of this article will be made available by the authors, without undue reservation.
