# Supplementary material for: A Simplified Amino Acidic Alphabet to Unveil the T-Cells Receptors Antigens: A Computational Perspective
Source: Front Chem. 2021 Feb 25;9:598802. doi: 10.3389/fchem.2021.598802 (PMC7947793; doi:10.3389/fchem.2021.598802)
Supplement: Supplementary file 1 [file datasheet1.docx]

A simplified amino acidic alphabet to unveil the T-Cells receptors antigens: a computational perspective

Raffaele Iannuzzi^1^, Grazisa Rossetti^2^, Andrea Spitaleri^3^, Raoul J.P. Bonnal^2^, Massimiliano Pagani^2,4^, Luca Mollica^4^*

^1^Istituto Nazionale Genetica Molecolare INGM 'Romeo ed Enrica Invernizzi', 20122 Milan, Italy.

^2^Molecular Oncology and Immunology, IFOM, via Adamello 16, 20139, Milan, Italy.

^3^Emerging Bacterial Pathogens Unit, Division of Immunology, Transplantation and Infectious Diseases, IRCCS San Raffaele Scientific Institute, via Olgettina 58, 20132, Milan, Italy.

^4^Department of Medical Biotechnology and Translational Medicine, University of Milan, Milan Italy

*** Correspondence:**Corresponding Author
[luca.mollica@unimi.it](mailto:luca.mollica@unimi.it)

Supplementary Material

# Supplementary Data

**Materials and methods**

***1. System preparation***

***1a. TCR structures editing***

Seven benchmarks have been chosen from the Protein Data Bank (PDB) for the present study: 1zgl (Y. Li et al., 2005), 2ian (Deng et al., 2007), 3mbe (Yoshida et al., 2010), 3t0e (Yin et al., 2012), 5ksb (Petersen et al., 2016), 6cqr (Galperin et al., 2018), 6dfx (Wang et al., 2019). They all have been edited in order to have the same chain names for the TCR (α and β), the antigen and the MHC-II (α and β) and the same numbering for the antigen regardless its length, then superimposed using Visual Molecular Dynamics (VMD) 1.9.3 (Humphrey et al., 1996)using the sequentially and structurally conserved regions of the TCR. We then extracted the TCR structures in complex with the antigen: in particular, we only retained the Vα and Vβ regions (see Supplementary Figure S1) because they are the only ones involved in the interactions with the antigen (and we removed the Cα and Cβ regions of the TCR, that are relevant only for the interaction with the T-cell surface), thus being able to speed up the docking calculations (see further). We also removed crystallization water from the structures.

We have reconstructed the CDR1/2/3 loops structure from scratch using Rosetta TCR, using the standalone program and the web server (Gowthaman & Pierce, 2018) in order to test the ability of this software to correctly reconstruct the CDR1/2/3 loops in the context of the whole presented pipeline. Assuming the point of view of any experimentalist who is moving from sequencing experiments, we extracted the amino acid variable sequences of the given structures and used them as input for Rosetta TCR. The result was in almost perfect agreement with the experimental data, i.e. with a backbone RMSD of the whole structure always under 1 Å. For this reason, we decided to use in the next step of the work the original structure edited as mentioned above.

***1b. Peptide design***

Peptides with the same backbone structure of the benchmark antigens have been designed using the Python library PeptideBuilder (PyPI, from the Biopython repository)(Tien et al., 2013) on the basis of backbone solid angles Φ and Ψ distribution of the antigens in the crystallographic structures of all the deposited TCR -antigen - MHC-II complexes (Painter & Stern, 2012) that have been previously verified on the 8 structure we selected as benchmarks of the present study. Considering only the residues in close contact with TCR and the MHC-II interface that binds the CDR loops of the TCR, we have built 9-mers because this peptide length should be the minimum length required for being antigenic in structural terms. The angles (with a standard deviation of ±2 degrees) are distributed as follows:

| Residue | Φ | Ψ |
| --- | --- | --- |
| P4 | 0 | 145 |
| P5 | -102 | 170 |
| P6 | -120 | 128 |
| P7 | -123 | 125 |
| P8 | -80 | 145 |
| P9 | -53 | 145 |
| P10 | -75 | 135 |
| P11 | -80 | 110 |
| P12 | 145 | 0 |

The peptides have been designed in order to focus on the interaction between the sidechains of residues P3, P5 and P8 and the surface of TCR exposed towards the MHC II, i.e. the cavities formed by the CDR3 loops. Hence we generated a general combinatorial library of peptides using the 6 amino acids that form the SCAA (according to criteria explained in the main text) for each of the aforementioned position and reducing the possibility of interaction between the peptide and the TCR using only fixed geometry glycines (G) for the other positions in order to exclude interactions with the TCR surface. This led to the generation of 6 x 6 x 6 = 216 different peptides in the form GGXGXGGXG, with X representing one of the 6 residues that constitute the SCAA.

***2. Rigid body docking calculations***

The HADDOCK software (Dominguez et al., 2003) consists of a collection of Python and CNS (Crytallography and NMR System) (Brünger et al., 1998) scripts with additional tools written in various languages. CNS is used as the computational engine that performs the computationally intensive part such as the energy calculations, minimizations and molecular dynamics refinement stages, while the Python routines are used for controlling the dataflow, scoring and performing various pre- and post-processing tasks.

The HADDOCK package has been downloaded alongside its license and installed on local machines equipped with Intel Core i3-8100T Quad Core 3,6 GHz processors and 8 GB of DDR-RAM running Ubuntu Linux 16.04 operating systems. A local repository containing all the peptides that had to be docked on TCR surface has been prepared in order to feed the docking procedure iteratively, passing to HADDOCK a list of peptides to be docked constituted as described in section 1b.

Inter- and intramolecular energies are evaluated using full electrostatic and van der Waals energy terms with an 8.5 Å cut-off with a shifting function for the electrostatic energy and switching function between 6.5 and 8.5 Å for the van der Waals energy using the OPLS nonbonded parameters (Jorgensen et al., 1996): this treatment can be considered the best compromise between speed and accuracy of the method with respect to the original OPLS force field setup (Honorato et al., 2019) Due to the nature of the SCAA histidines have been considered protonated as by HADDOCK default set up. The docking protocol we have used in the present work consists of randomization of orientations and rigid body energy minimization of the TCR and of the peptides, i.e. the first of the three steps the constitute the full HADDOCK protocol for docking proteins together (including potential flexible refinement). In the randomization stage, the two partner proteins are positioned at 150 Å from each other in space and each protein is randomly rotated around its centre of mass. Rigid body energy minimization is then performed (i.e., each macromolecule involved in the binding is kept rigid, with the TCR considered as a whole body despite being a dimer):  first, four cycles of orientational optimization are performed in which each protein in turn is allowed to rotate to minimize the intermolecular energy function. Then both translations and rotations are allowed, and the two proteins are docked by rigid body energy minimization. During these steps the solvent has been treated implicitly using a dielectric constant of 10, considered the best value for representing the electrostatic internal behavior of proteins (L. Li et al., 2013). 100 complex conformations have been calculated for each peptide of the list obtained as described in paragraph 1b, all of them retained and analysed (see further) in order to take into account the maximum energetic and geometrical variability of the sampling in a completely unbiased fashion.

During the rigid body docking procedure 6 ambiguous restraints have been adopted, i.e. distance restraints based upon original geometries of interaction between the centre of mass (c.o.m.) CD3α/β loops backbone atoms and the antigen’s backbone atoms. In particular (see Figure 2 of the main text):

Cα (c.o.m. CDR3a) – Cα (P2)

Cα (c.o.m. CDR3a) – Cβ (P3)

Cα (c.o.m. CDR3a) – Cα (P4)

Cα (c.o.m. CDR3b) – Cα (P4)

Cα (c.o.m. CDR3b) – Cβ (P5)

Cα (c.o.m. CDR3b) – Cα (P6)

where the centre of mass is calculated manually for any benchmark (see section 1.1) and based on the length of the hypervariable loops CDR3α and CDR3β extrapolated from the sequence corresponding to the deposited PDB structure and based on their flanking sequences common to all the TCR sequences in the conserved regions, as used in principle to build models in ROSETTA-TCR. The distance restraint lower limit was set up to 6 Å, the upper limit to 9 Å. No random removal of restraints, due to their small number, was used. The force constant for the restraints was set up to 10 kcal mol^-1^.

***3. Analysis and graphics***

The graphs that are reported in the present work have been realized with Gnuplot 5.4.0 (July 2020 release), whereas the molecular graphics images have been realized with Visual Molecular Dynamics (VMD) 1.9.3 (Humphrey et al., 1996). The binding energy is extracted from the output of HADDOCK in PDB format. The analysis of RMSD and of peptide-TCR contacts have been performed using the *g_rms* and *g_mindist* commands of the GROMACS suite (Abraham et al., 2015; Berendsen et al., 1995). The reference for the RMSD analysis is the original antigen pose of the peptide on the surface of TCR (the original size of the peptide has been edited in order to refer to the same size of the peptide, i.e. a 9-mer). The number of contacts has been computed as the overall number of distances lower than 6 Å computed for all the atoms of the peptide and of the TCR. The distributions of contacts, binding energies and RMSD have been analysed using the *g_analyze* GROMACS program.

***4. Informatic implementation***

At the present stage of the work, the entire workflow has been implemented in a divide-and-conquer fashion using separated modular scripts in Python and/or native Bash environment for the Linux operating system for each step. In particular:

4.1. *TCR modelling*: a script that manages the ROSETTA TCR jobs both on local machines and on Rosetta Commons server and gives back the 3D structure in PDB format of the TCR plus the modelled CDR loops;

4.2. *Peptide building*: an original built-in sequence in the form GXXGXGGXG is cyclically transformed in a library of .pdb files, that are subsequently used in the docking phase, replacing the X-marked positions with the desired residues (in the present work the list of replaced residues is composed of Y, V, H, Q, D and S). The script is based on Python language and integrated with the Bash environment, it is based on the editable user’s interaction with software Peptide Builder (Tien et al., 2013). The script is of general purpose, i.e. angles, sequences and amino acids can be adapted on users’ needs;

4.3. *Docking*: a docking calculation is set up making use of the previously built peptides’ structures repository. The script is based on Python language and integrated with the Bash environment, it is based on the editable user’s interaction with software HADDOCK (Dominguez et al., 2003). The script is of general purpose, i.e. all the docking parameters can be adapted on users’ needs;

4.4. *Statistical analysis*: the outcome of the docking calculations is processed in order to extract the information that are relevant for the analysis, i.e. binding energies, peptide-TCR contacts, average values and standard deviations of these parameters, RMSD with respect to the reference (if available). Moreover, this module is able to extract directly a selection of the sequences/peptides that are within an interval of 2σs and centred on the average values of energies and contacts.

All the scripts are available upon request.

**References**

Abraham, M. J., Murtola, T., Schulz, R., Páll, S., Smith, J. C., Hess, B., & Lindah, E. (2015). Gromacs: High performance molecular simulations through multi-level parallelism from laptops to supercomputers. *SoftwareX*, *1*–*2*, 19–25. https://doi.org/10.1016/j.softx.2015.06.001

Berendsen, H. J. C., Berendsen, H. J. C., Van Der Spoel, D., & Van Drunen, R. (1995). Gromacs: A message-passing parallel molecular dynamics implementation. *COMP. PHYS. COMM*, *91*, 43--56. http://citeseerx.ist.psu.edu/viewdoc/summary?doi=10.1.1.123.3928

Brünger, A. T., Adams, P. D., Clore, G. M., Delano, W. L., Gros, P., Grossekunstleve, R. W., Jiang, J. S., Kuszewski, J., Nilges, M., Pannu, N. S., Read, R. J., Rice, L. M., Simonson, T., & Warren, G. L. (1998). Crystallography & NMR system: A new software suite for macromolecular structure determination. *Acta Crystallographica Section D: Biological Crystallography*, *54*(5), 905–921. https://doi.org/10.1107/S0907444998003254

Deng, L., Langley, R. J., Brown, P. H., Xu, G., Teng, L., Wang, Q., Gonzales, M. I., Callender, G. G., Nishimura, M. I., Topalian, S. L., & Mariuzza, R. A. (2007). Structural basis for the recognition of mutant self by a tumor-specific, MHC class II-restricted T cell receptor. *Nature Immunology*, *8*(4), 398–408. https://doi.org/10.1038/ni1447

Dominguez, C., Boelens, R., & Bonvin, A. M. J. J. (2003). HADDOCK: A protein-protein docking approach based on biochemical or biophysical information. *Journal of the American Chemical Society*, *125*(7), 1731–1737. https://doi.org/10.1021/ja026939x

Galperin, M., Farenc, C., Mukhopadhyay, M., Jayasinghe, D., Decroos, A., Benati, D., Tan, L. L., Ciacchi, L., Reid, H. H., Rossjohn, J., Chakrabarti, L. A., & Gras, S. (2018). CD4+ T cell–mediated HLA class II cross-restriction in HIV controllers. *Science Immunology*, *3*(24). https://doi.org/10.1126/sciimmunol.aat0687

Gowthaman, R., & Pierce, B. G. (2018). TCRmodel: High resolution modeling of T cell receptors from sequence. *Nucleic Acids Research*, *46*(W1), W396–W401. https://doi.org/10.1093/nar/gky432

Honorato, R. V., Roel-Touris, J., & Bonvin, A. M. J. J. (2019). MARTINI-Based Protein-DNA Coarse-Grained HADDOCKing. *Frontiers in Molecular Biosciences*. https://doi.org/10.3389/fmolb.2019.00102

Humphrey, W., Dalke, A., & Schulten, K. (1996). VMD: Visual molecular dynamics. *Journal of Molecular Graphics*, *14*(1), 33–38. https://doi.org/10.1016/0263-7855(96)00018-5

Jorgensen, W. L., Maxwell, D. S., & Tirado-Rives, J. (1996). *Development and Testing of the OPLS All-Atom Force Field on Conformational Energetics and Properties of Organic Liquids*.

Li, L., Li, C., Zhang, Z., & Alexov, E. (2013). On the dielectric “constant” of proteins: Smooth dielectric function for macromolecular modeling and its implementation in DelPhi. *Journal of Chemical Theory and Computation*, *9*(4), 2126–2136. https://doi.org/10.1021/ct400065j

Li, Y., Huang, Y., Lue, J., Quandt, J. A., Martin, R., & Mariuzza, R. A. (2005). Structure of a human autoimmune TCR bound to a myelin basic protein self-peptide and a multiple sclerosis-associated MHC class II molecule. *EMBO Journal*, *24*(17), 2968–2979. https://doi.org/10.1038/sj.emboj.7600771

Painter, C. A., & Stern, L. J. (2012). Conformational variation in structures of classical and non-classical MHCII proteins and functional implications. *Immunological Reviews*, *250*(1), 144–157. https://doi.org/10.1111/imr.12003

Petersen, J., Kooy-Winkelaar, Y., Loh, K. L., Tran, M., van Bergen, J., Koning, F., Rossjohn, J., & Reid, H. H. (2016). Diverse T Cell Receptor Gene Usage in HLA-DQ8-Associated Celiac Disease Converges into a Consensus Binding Solution. *Structure*, *24*(10), 1643–1657. https://doi.org/10.1016/j.str.2016.07.010

Tien, M. Z., Sydykova, D. K., Meyer, A. G., & Wilke, C. O. (2013). PeptideBuilder: A simple Python library to generate model peptides. *PeerJ*, *1*(1), e80. https://doi.org/10.7717/peerj.80

Wang, Y., Sosinowski, T., Novikov, A., Crawford, F., White, J., Jin, N., Liu, Z., Zou, J., Neau, D., Davidson, H. W., Nakayama, M., Kwok, W. W., Gapin, L., Marrack, P., Kappler, J. W., & Dai, S. (2019). How C-terminal additions to insulin B-chain fragments create superagonists for T cells in mouse and human type 1 diabetes. *Science Immunology*, *4*(34). https://doi.org/10.1126/sciimmunol.aav7517

Yin, Y., Wang, X. X., & Mariuzza, R. A. (2012). Crystal structure of a complete ternary complex of T-cell receptor, peptide-MHC, and CD4. *Proceedings of the National Academy of Sciences of the United States of America*, *109*(14), 5405–5410. https://doi.org/10.1073/pnas.1118801109

Yoshida, K., Corper, A. L., Herro, R., Jabri, B., Wilson, I. A., & Teyton, L. (2010). The diabetogenic mouse MHC class II molecule I-Ag7 is endowed with a switch that modulates TCR affinity. *Journal of Clinical Investigation*, *120*(5), 1578–1590. https://doi.org/10.1172/JCI41502

# Supplementary Figures


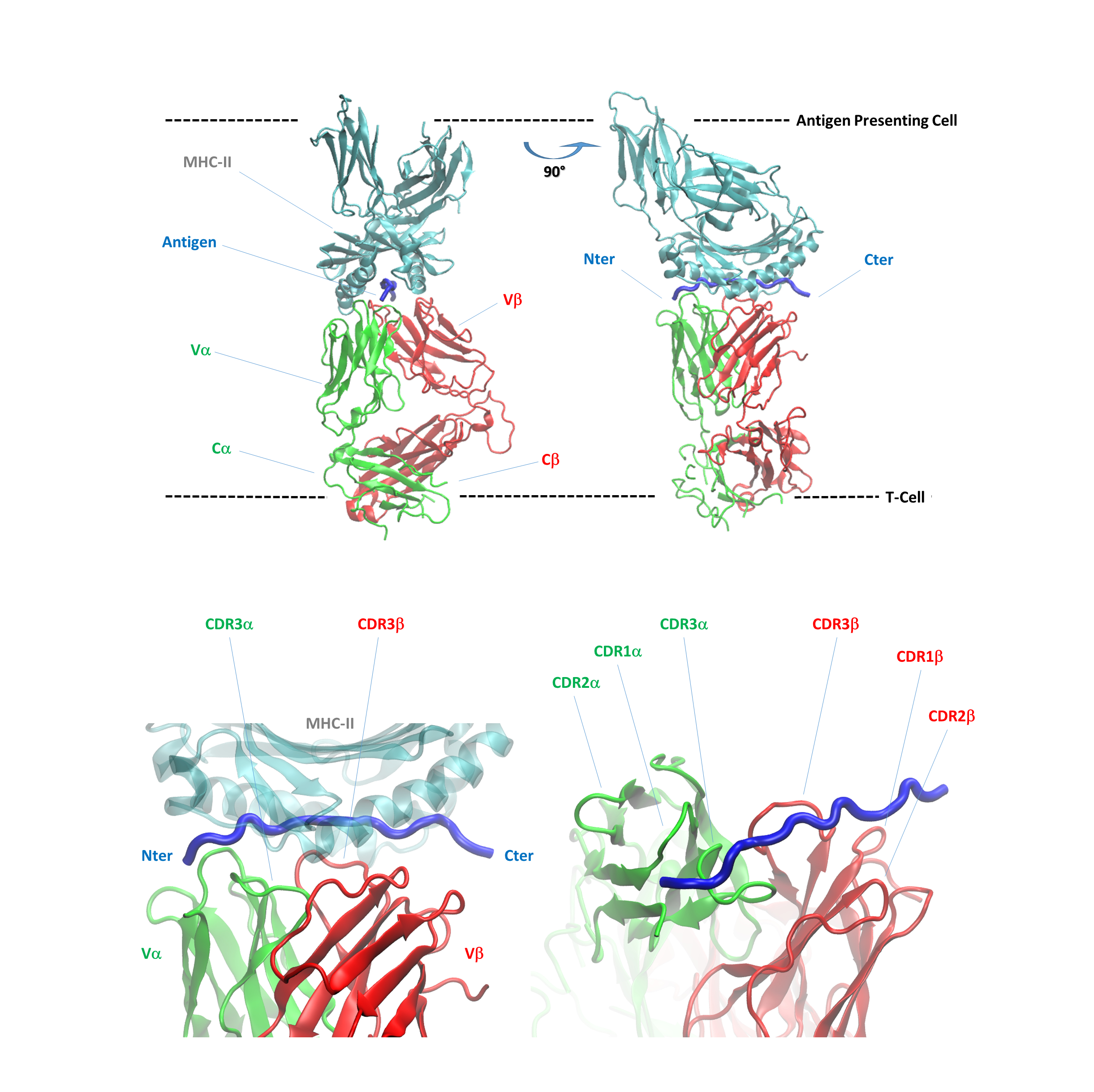


**Supplementary Figure S1**: General scheme of the complex formed by MHC-II, antigen and TCR. The antigen is represented in blue, the α chain of the TCR in green, the β chain of the TCR in red.


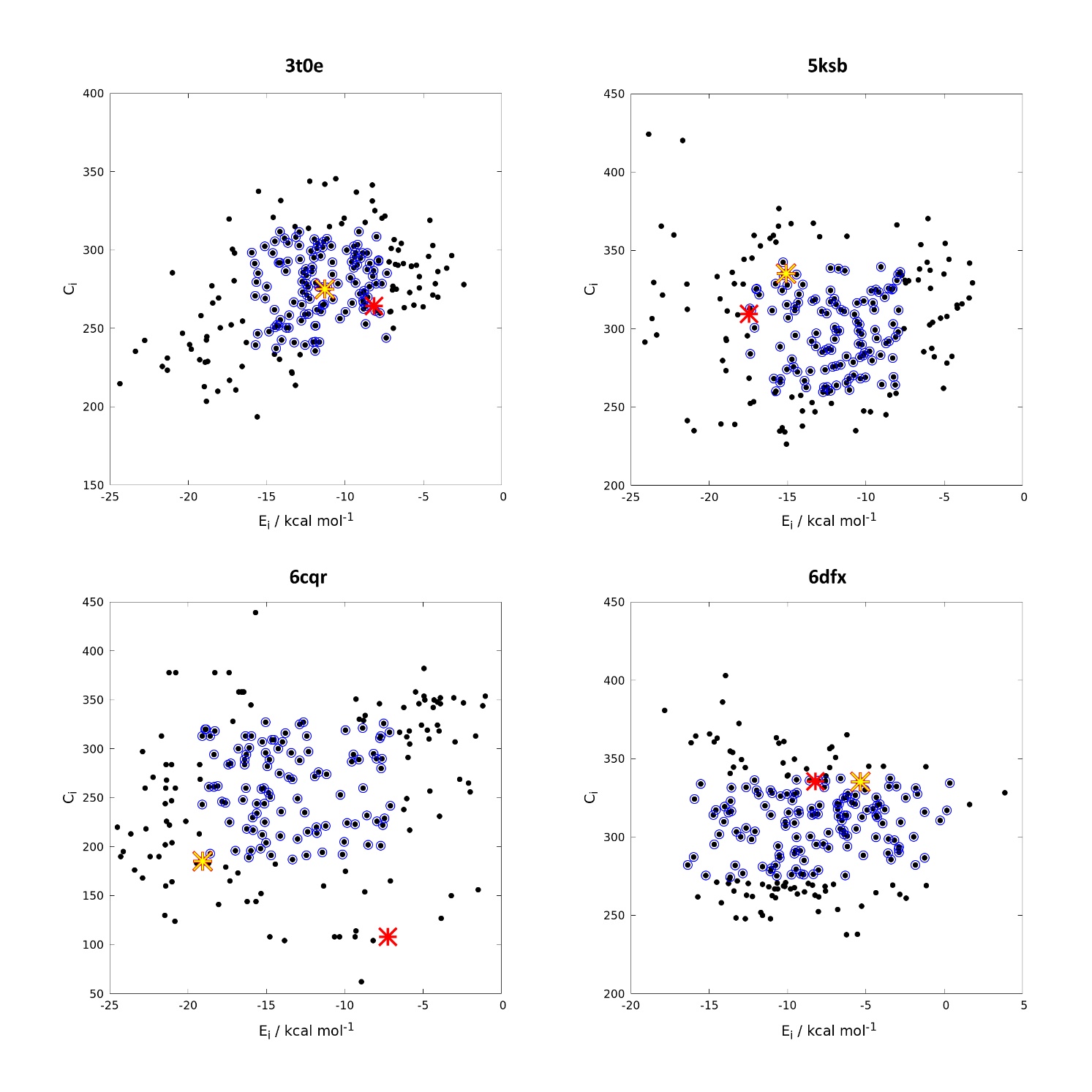


**Supplementary Figure S2**: Energy vs contact maps relative to the benchmarks 3t0e, 5ksb, 6cqr, 6dfx. Every black dot represents the average of 100 poses for a single peptide; the blue dots correspond to values of energies, E_i_, and contacts, C_i_, that fall within intervals of ±σ centered with respect to average values of the plotted property; the red cross corresponds to the original triad of amino acids in positions P3, P5 and P8; the yellow star corresponds to the translated triad of amino acids in positions P3, P5, P8.


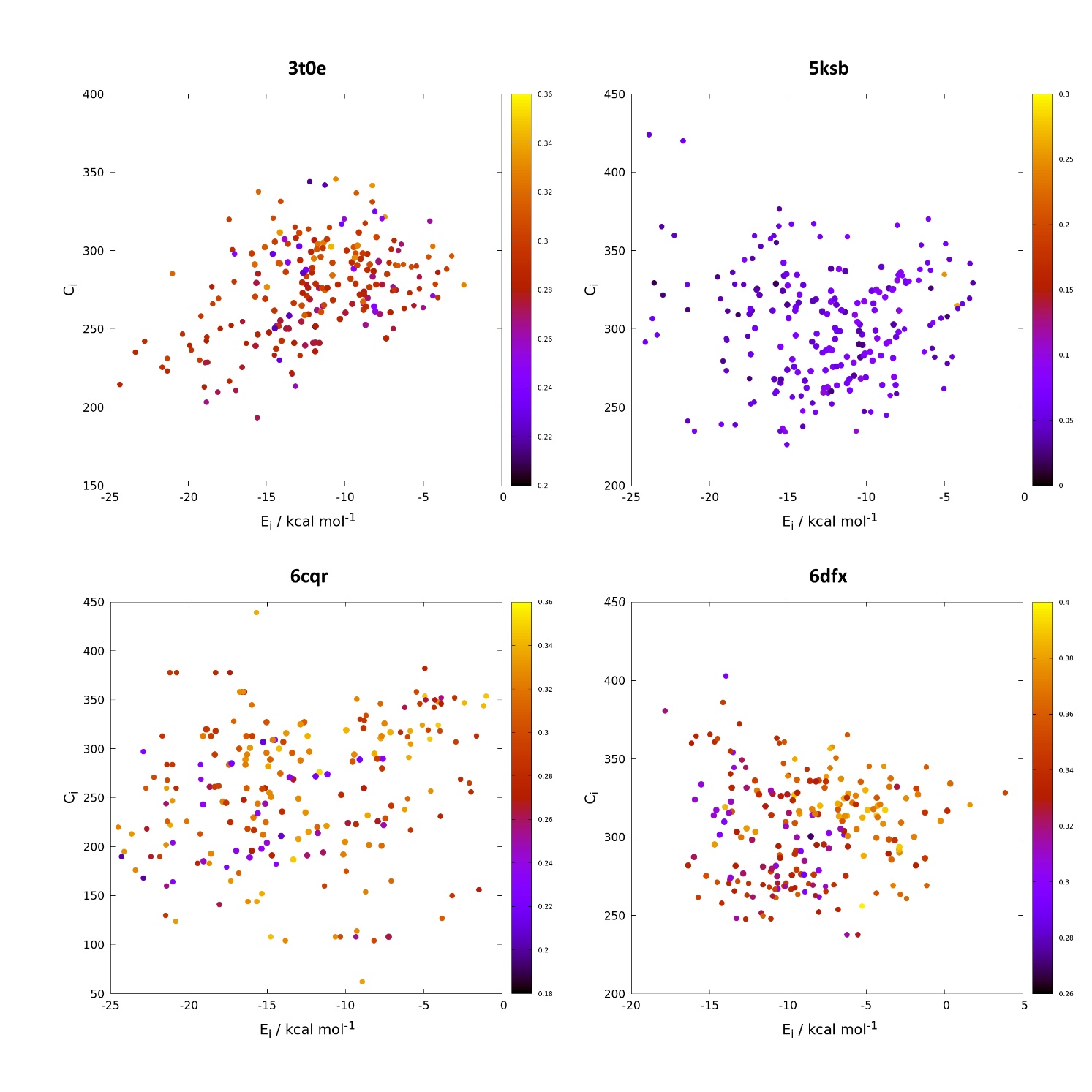


**Supplementary Figure S3**: Energy vs contact maps relative to the benchmarks 3t0e, 5ksb, 6cqr, 6dfx: every dot is coloured according to the RMSD (expressed in nm) with respect to the original experimental position.

**
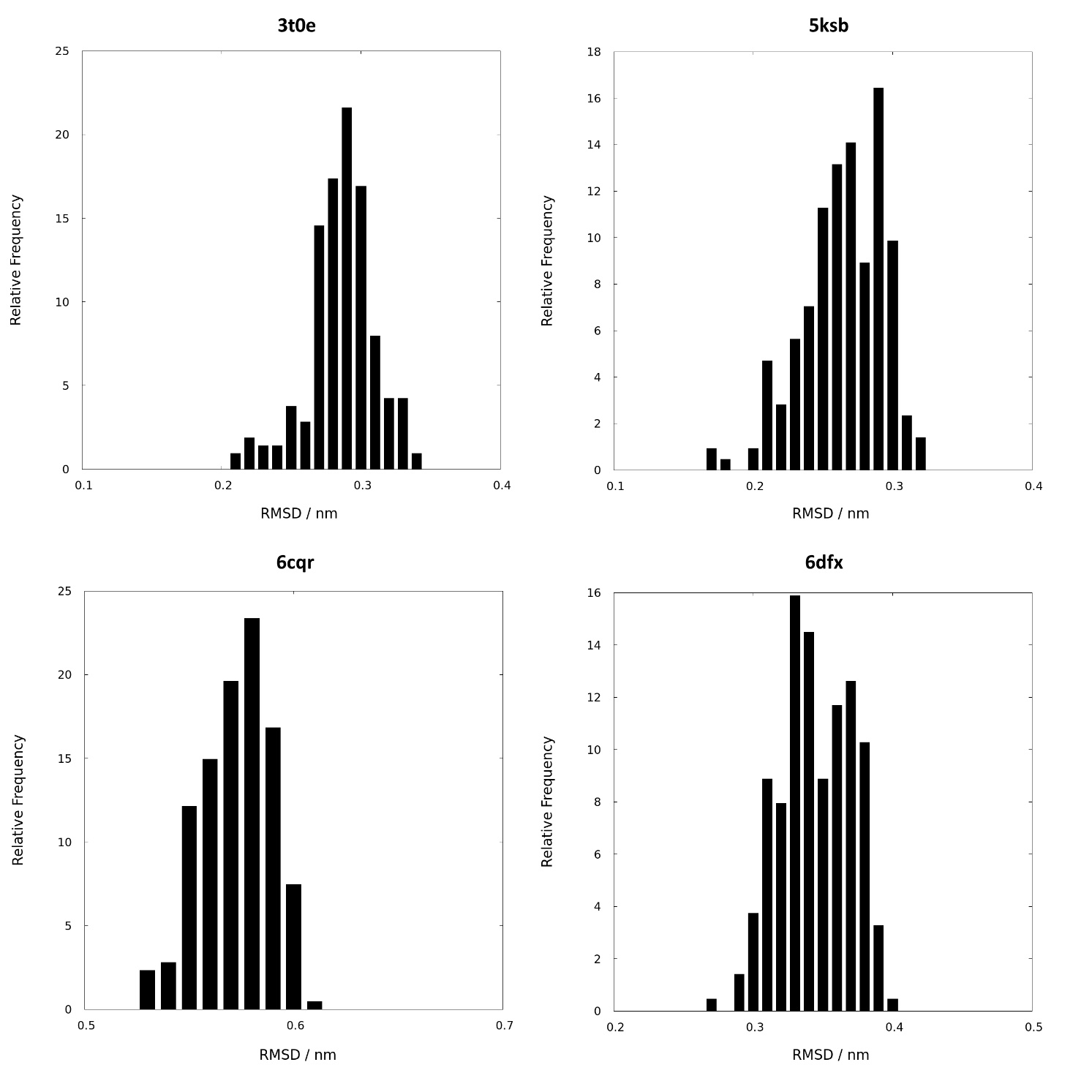
**

**Supplementary Figures S4**: RMSD distribution of the peptides for 3t0e, 5ksb, 6cqr, 6dfx benchmarks.

**
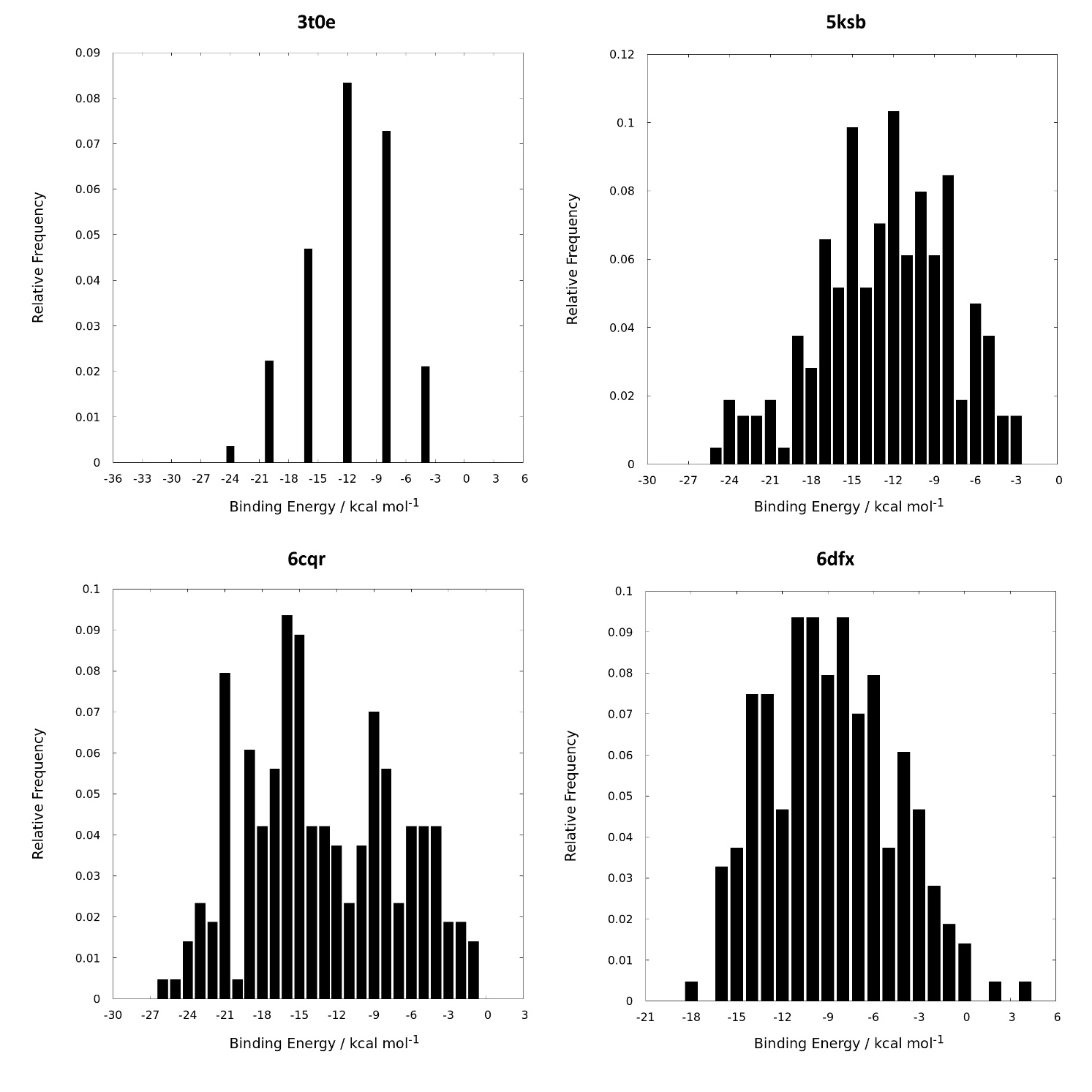
**

**Supplementary Figures S5**: E_i_ of the peptides for 3t0e, 5ksb, 6cqr, 6dfx benchmarks.


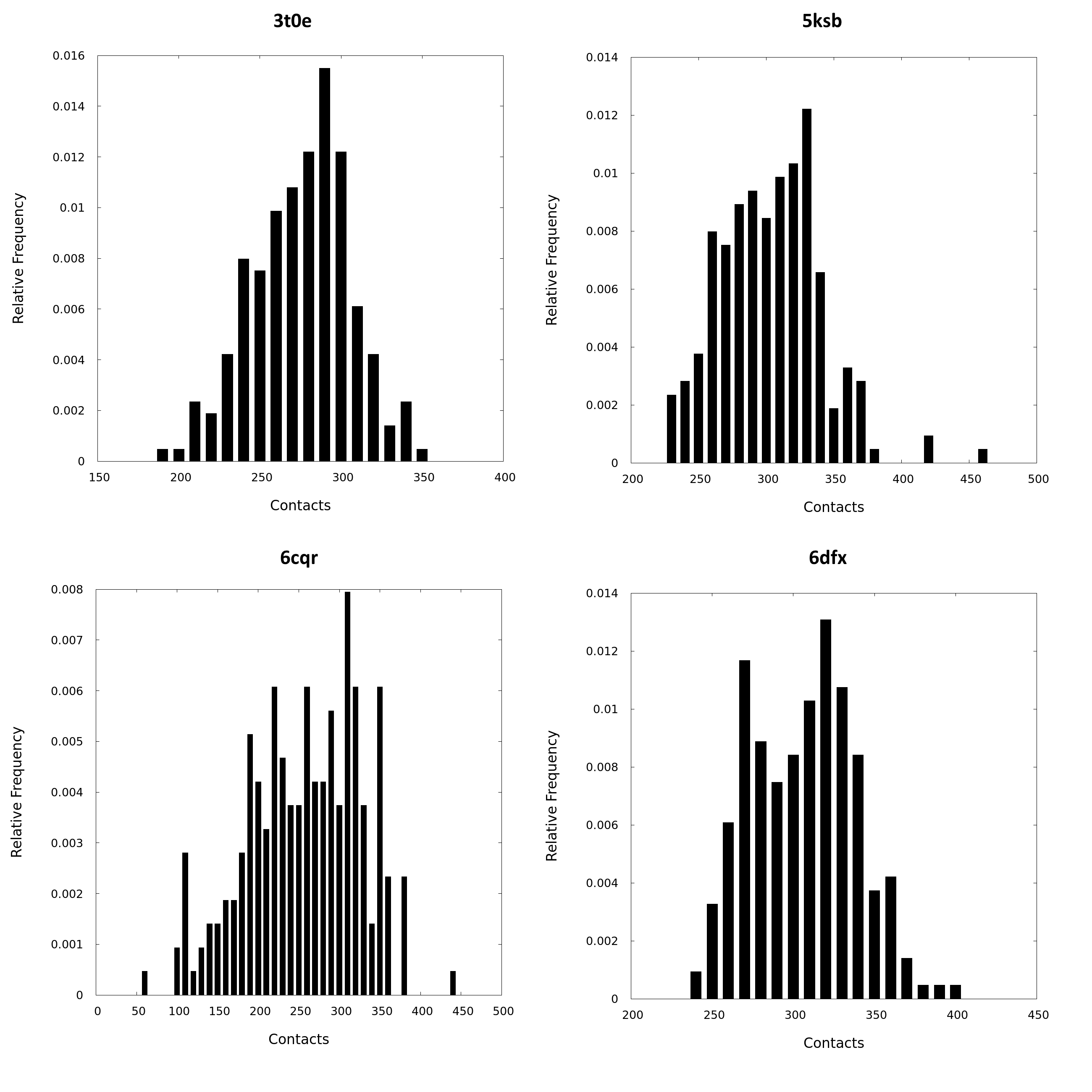


**Supplementary Figures S6**: C_i_ of the peptides for 3t0e, 5ksb, 6cqr, 6dfx benchmarks.

**
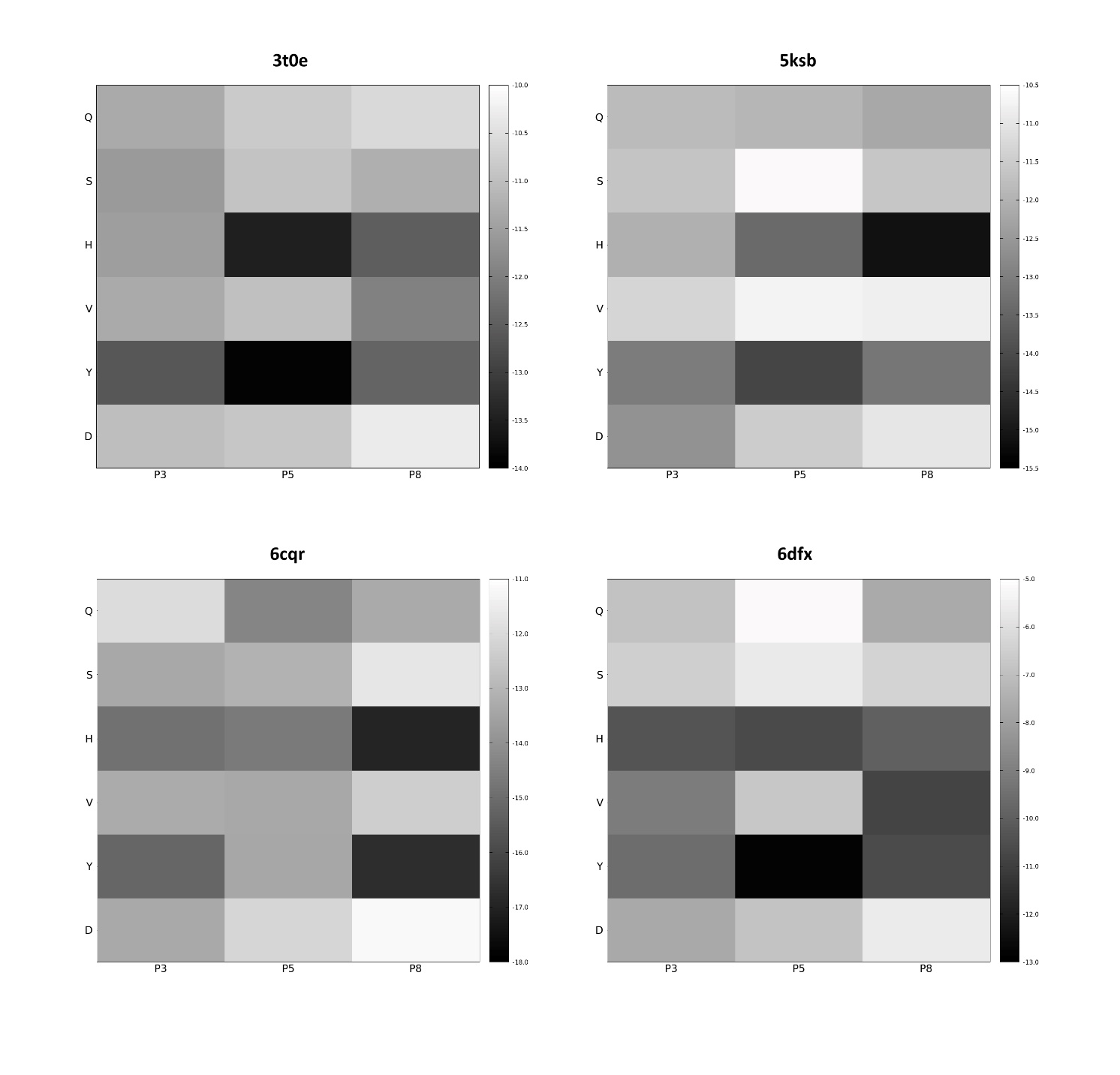
**

**Supplementary Figure S7**: Energy residual content (expressed in kcal mol^-1^) heatmaps for the benchmarks 3t0e, 5ksb, 6cqr, 6dfx with respect to the SCAA class (vertical axis) and the three positions along the antigen sequence P3, P5 and P8 (horizontal axis).


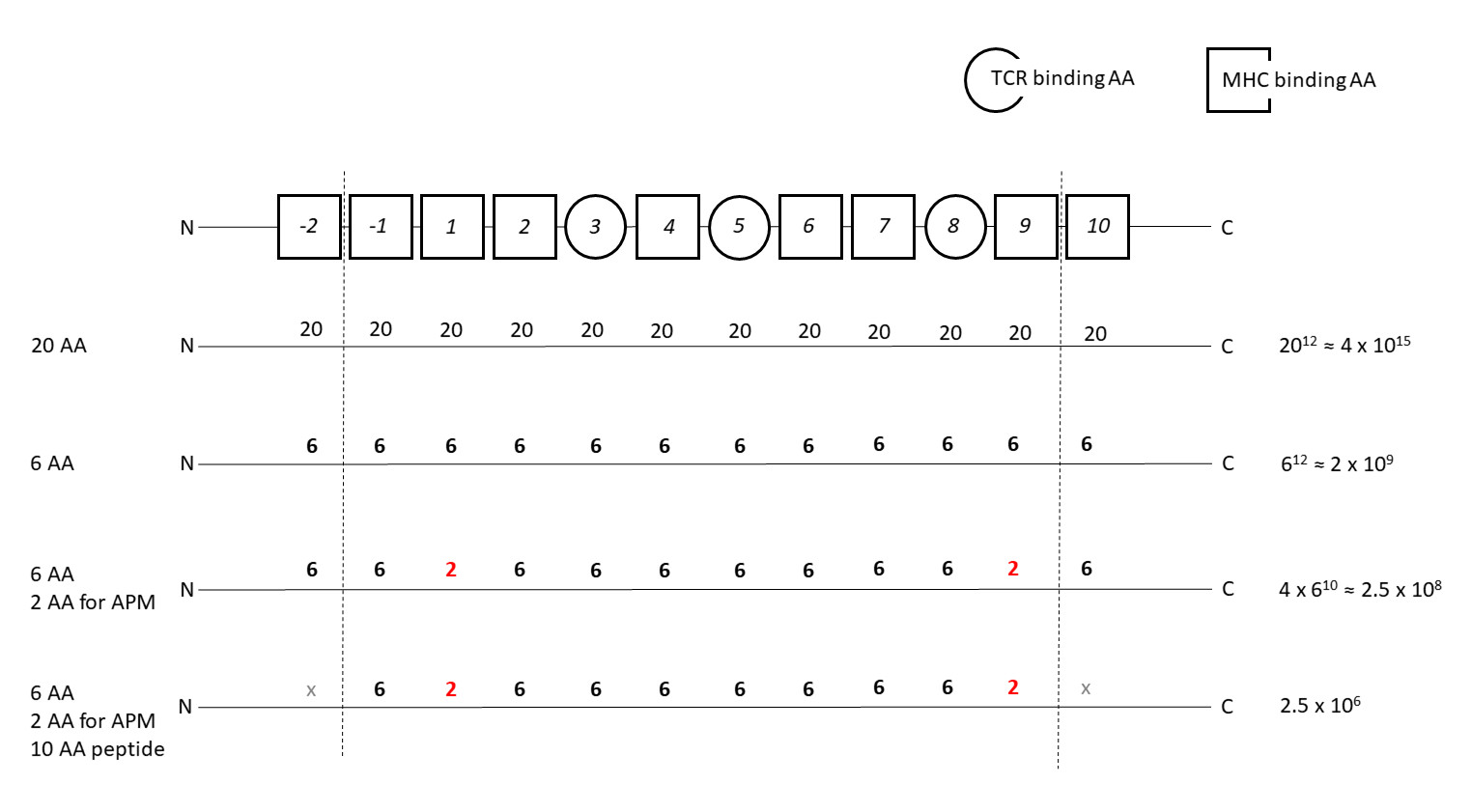


**Supplementary Figure S8**: general dimensionality reduction scheme for the construction of the SCAA. On the right: the number of possible combinations given an ensemble of amino acids in a given position (APM: anchoring point for the MHC II). From the top to the bottom: 20 amino acids (AA) for each position, 6 AA for each position, 6 AA for each position but 2 for the APM; further reduction of the peptide length to 10 AA.

| **PDB/SA** | **Refe­rence triad** | **E_i_ ref / kcal mol^-1^** | **C_i_ ref** | **E_i_ min/ kcal mol^-1^** | **E_i_ max/ kcal mol^-1^** | **C_i_ Min** | **C_i_ max** | **Translated triad** | **E_i_/ kcal mol^-1^** | **C_i_** |
| --- | --- | --- | --- | --- | --- | --- | --- | --- | --- | --- |
| 1zgl | N V R | -12.6 | 298 | -20.1 | -10.8 | 241 | 329 | Q V H | -18.8 | 282 |
| SA1 | L P K | -17.1 | 251 |  |  |  |  | V V H | -19.6 | 291 |
| SA2 | L L K | -12.6 | 244 |  |  |  |  | V V H | -19.6 | 291 |
| SA3 | L T K | -15.2 | 254 |  |  |  |  | V S H | -19.8 | 310 |

Supplementary Table T1: List of superantigens tested for the 1zgl structure in the present work as reported in (Y. Li et al., 2005). SA1, SA2 and SA3 indicate respectively the triads of original antigens of general sequence (numbered from P1) FKLIXTYKZ with P3 = L, P5 = X = L/T/P and P8 = K (the other residues have been translated into G, as explained in the main text). The first line has been reported from Table 1 in the main text for comparison. The original triad of TCR binding amino acids and its translation in the SCAA are reported together with binding energy values E_i_ and the number of contacts C_i_ with the TCR respectively in in kcal mol^-1^ and as pure numerical values.
